# A novel cell membrane affinity sample pretreatment technique for recognition and preconcentration of active components from traditional Chinese medicine

**DOI:** 10.1038/s41598-017-03709-6

**Published:** 2017-06-15

**Authors:** Yusi Bu, Xiaoshuang He, Qi Hu, Cheng Wang, Xiaoyu Xie, Sicen Wang

**Affiliations:** 1 School of Pharmacy, Health Science Center, Xi’an Jiaotong University, Xi’an, 710061 China; 2 Shaanxi Engineering Research Center of Cardiovascular Drugs Screening & Analysis, Xi’an, 710061 China

## Abstract

We describe a novel biomembrane affinity sample pretreatment technique to quickly screen and preconcentrate active components from traditional Chinese medicine (TCM), which adopts cell membrane coated silica particles (CMCSPs) as affinity ligands which benefit the biomembrane’s ability to maximize simulation of drug-receptor interactions *in vivo*. In this study, the prepared CMCSPs formed by irreversible adsorption of fibroblast growth factor receptor 4 (FGFR4) cell membrane on the surface of silica were characterized using different spectroscopic and imaging instruments. Drug binding experiments showed the excellent adsorption rate and adsorption capacity of FGFR4/CMCSPs compared with non-coated silica particles. The FGFR4/CMCSPs were used as solid-phase extraction sorbents to pretreat the TCM *Aconitum szechenyianum Gay*. The resultant FGFR4/CMCSPs exhibited good performance. In addition, high selectivity and recognition ability of the FGFR4/CMCSPs were determined by selectivity experiments. Four alkaloid were screened and identified, one of these alkaloid, napellonine, showed favorable anti-tumor activity in preliminary pharmacological verification trials including cell proliferation and molecular docking assays. The proposed cell membrane affinity sample pretreatment method is a reliable, effective and time-saving method for fast screening and enriching active compounds and can be extended to pretreat other TCMs as leading compounds resources.

## Introduction

Solid-phase extraction (SPE)^1^, a popular sample pretreatment technique, has been developed and widely used due to its high speed^2^, availability^3, 4^, simplicity^5, 6^ and high preconcentration factors. However, common sorbents such as C_18_ have low selectivity, which has prevented its wide application in screening specific species. As the type of sorbent is vital to analytical methods, exploring novel sorbents for screening and concentrating is necessary for discovering potential drugs.

In recent years, biological materials such as enzymes, proteins and biomembranes, which can be used as targets to screen specific species in chromatographic analysis^7–11^, SPE^12–14^ and biosensors^15^, have become a research hotspot. Modern pharmaceutical studies have shown that of all drug actions *in vivo*, the most important is drug binding with its receptors on the cell membrane^16–18^. Receptor pharmacology has demonstrated that biological responses are activated for the initiation of signal transduction when drugs interact with cell membrane receptors, and consequently result in special pharmacological actions. Thus, the cell membrane is an effective tool to investigate the action mechanism and screen active drugs^19, 20^. To this end, we designed novel cell membrane coated silica particles (CMCSPs) which not only recognized but also preconcentrated bioactive compounds from traditional Chinese medicine (TCM).

TCMs are natural therapeutic remedies and are attracting significant attention due to their clinical use and reliable treatment effects. They can be regarded as a source of leading compounds for new drug discovery^21, 22^. Alkaloids from the genus *Aconitum* have been research hotspots due to their antitumor activity^23, 24^. *Aconitum szechenyianum Gay* (ASG) belonging to the genus *Aconitum* has been used in northwest China for more than 500 years. Pharmacological studies demonstrate that it can be used for expelling wind, removing dampness and has anti-inflammatory, analgesic and antitumor activity^25–27^. However, the screening and analysis of bioactive components from TCMs have proven difficult due to their complex chemical compositions and low content of active ingredients. In addition, it is considered that of the hundreds of chemicals in TCMs, only active compounds are associated with clinical efficacy, while others are ineffective or even accentuate illness^28^. Thus, recognization and confirmation of active components are very important in order to clarify pharmacologic mechanisms and ensure the effectiveness and safety of drug use.

Fibroblast growth factors receptor 4 (FGFR4), belongs to FGFR family, which are members of the tyrosine kinase receptor family, is composed of a three-extracellular immunoglobulin-like domain, a transmembrane domain and a tyrosine kinase domain and plays a significant role in tumorigenesis^29, 30^. Clinical studies have shown that the over-expression of FGFR4 is related to many malignant tumors such as melanoma, prostate cancer^31^, gastric cancer^32^ and liver cancer^33, 34^. In recent years, the extracellular and intracellular domains of FGFR4 have been investigated as novel targets for drug discovery and some FGFR4 inhibitors such as PD173074 (PD) have been widely used in clinical research^32, 35^. Therefore, FGFR4 may be a potential drug target for screening anti-tumor leading compounds.

**Figure 1:**
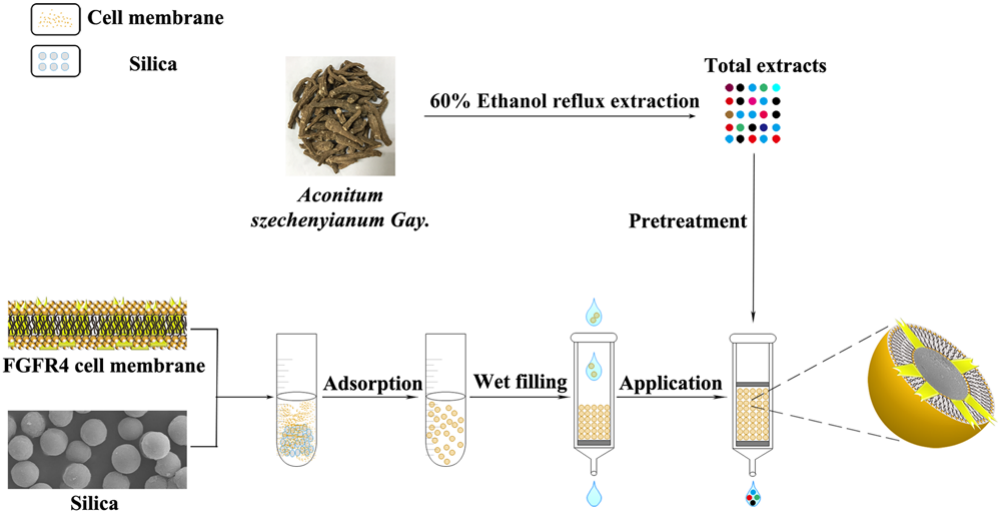
Schematic illustration of the procedures of FGFR4/CMCSPs SPE system.

In the present study, a novel FGFR4/CMCSPs sample pretreatment method was designed and schematically depicted in Fig. 1 to specifically screen and preconcentrate bioactive components from TCM. Scanning electron microscopy (SEM), energy dispersive spectrometry (EDS), thermo gravimetric analysis (TGA) and differential scanning calorimetry (DSC) were used to characterize the biomaterials. The adsorption capacity and adsorption kinetics of CMCSPs and non-coated silica particles (NCSPs) were investigated. The obtained materials were used as SPE sorbents coupled with high performance liquid chromatography/time-of-flight mass spectrometry (HPLC/TOFMS) to determine the leading compounds in ASG. Moreover, cell proliferation and molecular docking tests were conducted to confirm the biological activities of the screened active components.

## Results and Discussion

### Preparation and Characterization of FGFR4/CMCSPs

To obtain more effective CMCSPs, the amount of cell membrane on the surface of the CMCSPs, determined by the amount of cell in the preparation, was further optimized. The optimum amount of cell for preparing biomembrane to coat silica was chosen as 1 × 10^7^ per 5 mg silica gel which reached saturation with an adsorption efficiency of 75% (Table 1).

**Table 1:** Sorbents Compositions of Cell Membrane Bound to Silica Gel^a^.

Sorbents	Silica gel (mg)	Cells (1 × 10^5^)	Bound cell membrane (%)
CMCSPs-1	5.0	1	100 ± 1.5
CMCSPs-2	5.0	10	100 ± 1.8
CMCSPs-3	5.0	100	75 ± 4.5
CMCSPs-4	5.0	200	31.25 ± 3.4
CMCSPs-5	5.0	300	33 ± 4.1

^a^Data are shown as mean ± RSD.

Based on the results from the optimization of preparation conditions, a specific adsorption mechanism is discussed. According to the literature^36^, silica is a common stationary phase in sample pretreatment which has polar silanol groups (Si-OH) on the surface. The bond between silica and the cell membrane aimed to take advantage of the irreversible adsorption properties of silica and the self-fusion characteristic of the cell membrane. The optimized amount of cell membrane could completely cover the silica surface due to the special self-fusion characteristic of the biomembrane without exposing the residual silanol groups. Ionic interactions, hydrophobic interactions and hydrogen bonding of the receptors on cell membranes may clarify the specific bonds between receptors and their positive drug.

**Figure 2:**
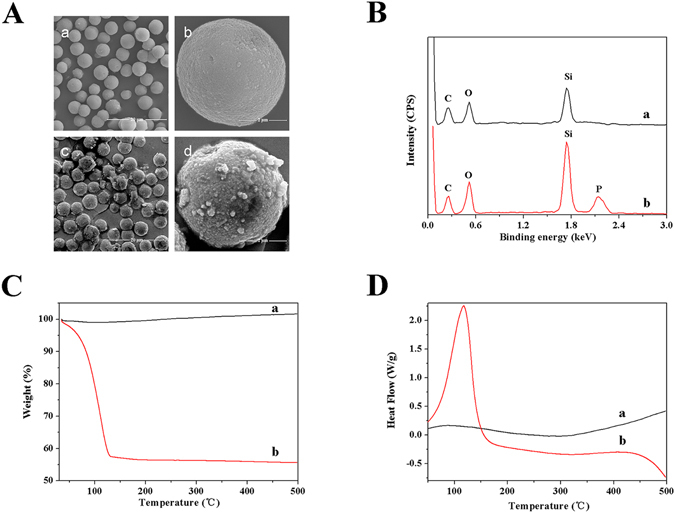
Characterizations of (**A**) SEM images of NCSPs (a and b) and CMCSPs (c and d); (**B**) EDS curves of NCSPs (a) and CMCSPs (b); (**C**) TGA images of NCSPs (a) and CMCSPs (b); (**D**) DSC results of NCSPs (a) and CMCSPs (b).

SEM was performed to observe the morphological features of CMCSPs and NCSPs. It was observed that the FGFR4/CMCSPs had a non-smooth membrane surface (Fig. 2Ad), while the surface of NCSPs was smooth (Fig. 2Ab). In addition, both the FGFR4/CMCSPs (Fig. 2Ac) and NCSPs (Fig. 2Aa) were distributed uniformly. Thus, after adsorption on the cell membrane, CMCSPs retained their spherical morphology. These findings suggested that the silica surface was successfully covered by the FGFR4 cell membrane.

To further clarify the adsorption of cell membrane on the surface of silica, EDS was chosen to characterize the chemical composition of the surface of CMCSPs and NCSPs. As shown in Fig. 2B, characteristic peaks for carbon, oxygen and silicon were clearly detected at 0.282, 0.523 and 1.740 keV, respectively. An obvious peak of phosphorus at 2.013 keV in FGFR4/CMCSPs was observed (Fig. 2Bb) compared with NCSPs (Fig. 2Ba), suggesting that the silica surface was covered by cell membrane.

TGA and DSC analyses were performed to further investigate the mass ratio between adsorbed cell membrane and its support materials, silica particles. The TGA and DSC results for FGFR4/CMCSPs and NCSPs are shown in Fig. 2C and D. The thermal decomposition of the two samples took place at a programmed temperature of 35–500 °C, and showed that the TGA curve of NCSPs was almost a straight line with no impurities (Fig. 2Ca). In the case of FGFR4/CMCSPs, almost half of the weight loss occurred between 35 and 130 °C (Fig. 2Cb) and was associated with dehydration of the cell membrane coated on silica, which confirmed that the cell membrane had been successfully coated on the silica surface. In addition, the results of thermal analysis by DSC proved that the endothermal peak (35–130 °C) was due to decomposition of the cell membrane (Fig. 2Db). The remaining silica gel showed good thermal stability similar to the NCSPs.

### Adsorption Capacity of FGFR4/CMCSPs

**Figure 3:**
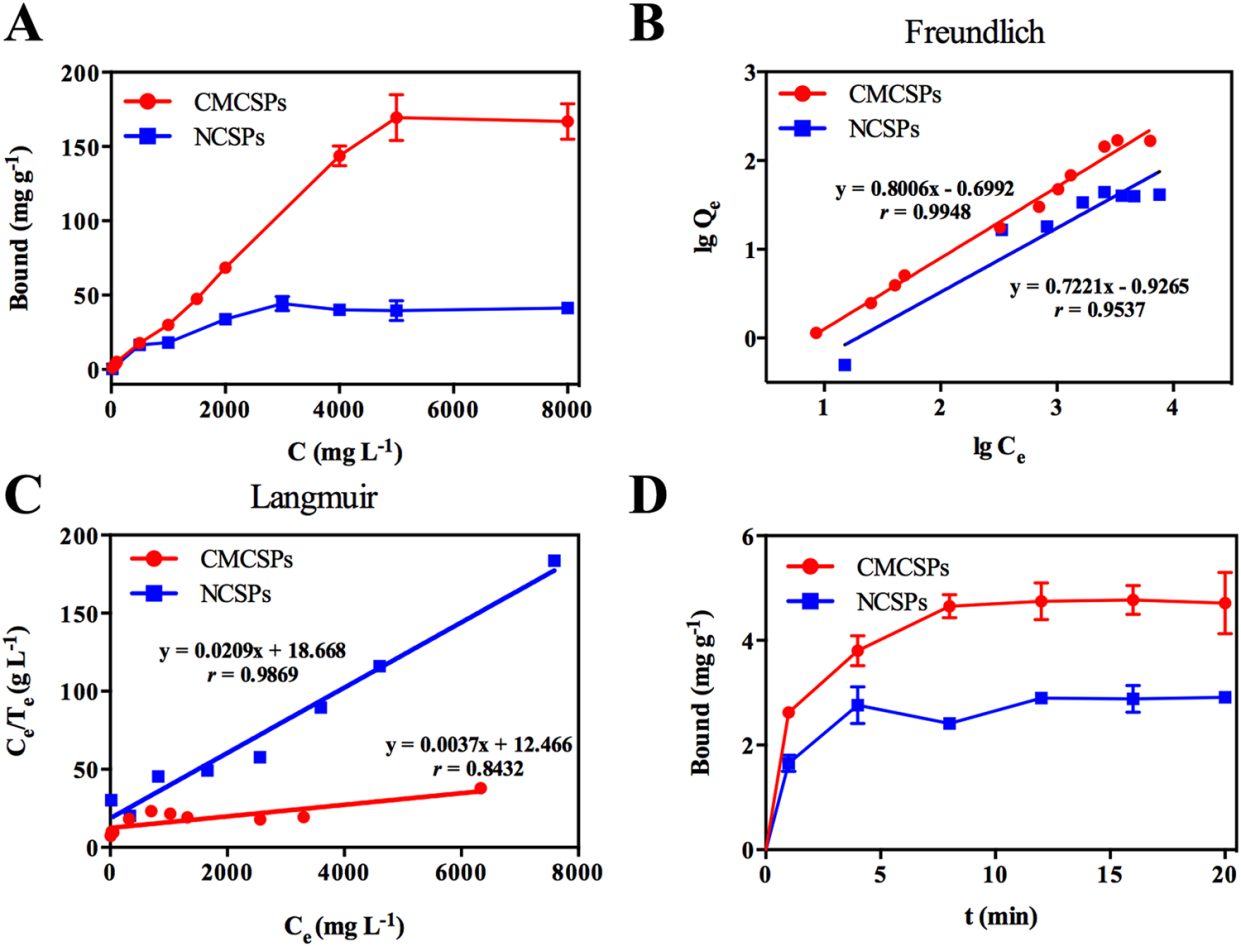
Binding properties and adsorption isotherm fitting of the FGFR4/CMCSPs and NCSPs. (**A**) Static adsorption isotherms; (**B** and **C**) adsorption isotherm fitting models; (**D**) kinetics adsorption.

The adsorption capacity was a significant factor when evaluating the FGFR4/CMCSPs. It was shown that the adsorbed amount increased quickly when the initial amount of PD increased (Fig. 3A), and when the initial concentration of PD on FGFR4/CMCSPs and NCSPs reached 5000 and 3000 mg L^−1^, the static adsorption capacity saturated and reached 169.52 and 44.28 mg g^−1^, respectively. These results suggested that the FGFR4 receptor binding sites were almost completely occupied by positive drug, indicating that the FGFR4/CMCSPs had satisfactory binding capacity for PD, compared to NCSPs.

In order to further evaluate the binding properties of FGFR4/CMCSPs, the Langmuir isotherm (LI) and Freundlich isotherm (FI) were adopted to analyze the obtained data. From the results shown in Fig. 3 and Table 2, it can be seen that the FGFR4/CMCSPs can be accurately modeled by the FI (*r* = 0.9948) (Fig. 3B), while the LI suitably described the static adsorption of NCSPs with a satisfactory correlation coefficient (*r* = 0.9869) (Fig. 3C)^37–40^. The FI demonstrated heterogeneous adsorption. In the FI, the Freundlich constants *K*_*F*_ and *m* represent the adsorption capacity and adsorption intensity, respectively. High *K*_*F*_ represented greater adsorption capacity. *M* varied between 0 and 1 and increased with decreased heterogeneity of the materials. Based on the FI which accurately modeled the FGFR4/CMCPs, the materials showed a heterogeneous distribution of binding sites with heterogeneity indices (*m*) of 0.801 due to the non-uniformly distributed FGFR4 receptors on the surface of silica. For NCSPs, the *r* value in the LI was larger than that in the FI which indicated that the LI was a suitable fit for the obtained data. The LI is a widely used adsorption isotherm and suggests monolayer adsorption. In the LI, adsorption sites are distributed homogeneously and adsorbed molecules do not interact with each other. Therefore, the perfect match of NCSPs on the LI model suggested that binding sites were distributed homogeneously due to hydroxyl groups evenly distribution on the surface of silica. This is consistent with previous SEM characterization (Fig. 2A), which showed that CMCSPs tend to be more heterogeneous than NCSPs.

**Table 2:** Static Adsorption Fitting Data of the CMCSPs and NCSPs.

Isotherm model	Equation	Parameter	CMCSPs	NCSPs
Langmuir^a^	*C*_*e*_/*Q*_*e*_ = (1/*Q*_*max*_) *C*_*e*_ + 1/(*K*_*L*_ *Q*_*max*_)	*R*	0.8432	0.9869
		*Q*_*max*_ (mg g^−1^)	357.142	47.847
		*K*_*L*_ (L g^−1^)	0.214 × 10^−3^	1.12 × 10^−3^
Freundlich^b^	*lgQ*_*e*_ = *mlgC*_*e*_ + lg*K*_*F*_	*R*	0.9948	0.9537
		*K*_*F*_ (mg g^−1^)	0.200	0.118
		*m*	0.801	0.722

^a^ *Q*_*e*_ (mg g^−1^) is the binding capacity; *C*_*e*_ (mg L^−1^) is equilibrium concentration in the solution; *Q*_*max*_ (mg g^−1^) is the theoretical maximum adsorption amount; *K*_*L*_ (L mg^−1^) is the adsorption equilibrium constant.

^b^ *K*_*F*_ is a Freundlich constant related to the adsorption capacity; *m* is a constant representing the adsorption intensity or surface heterogeneity.

### Adsorption Kinetics of FGFR4/CMCSPs

The adsorption kinetics of CMCSPs are a major influence on fast screening and separation in practical applications. Figure 3D shows the absorption kinetics results. The adsorption amount of PD on the FGFR4/CMCSPs increased rapidly in the first 8 min. As the binding period increased, the binding amount slowly increased and finally reached equilibrium after 12 min when the binding amount was almost 4.74 mg g^−1^. The equilibrium time showed that the recognition process between FGFR4 receptors on the cell membrane and positive drug was reasonably good, which led to easier binding of active compounds during the actual application. The adsorption kinetics of NCSPs were also investigated. With increased time, binding amount of PD was very low compared to CMCSPs, demonstrating the high selectivity of CMCSPs due to FGFR4 receptors on the cell membrane.

### Selectivity of FGFR4/CMCSPs

**Figure 4:**
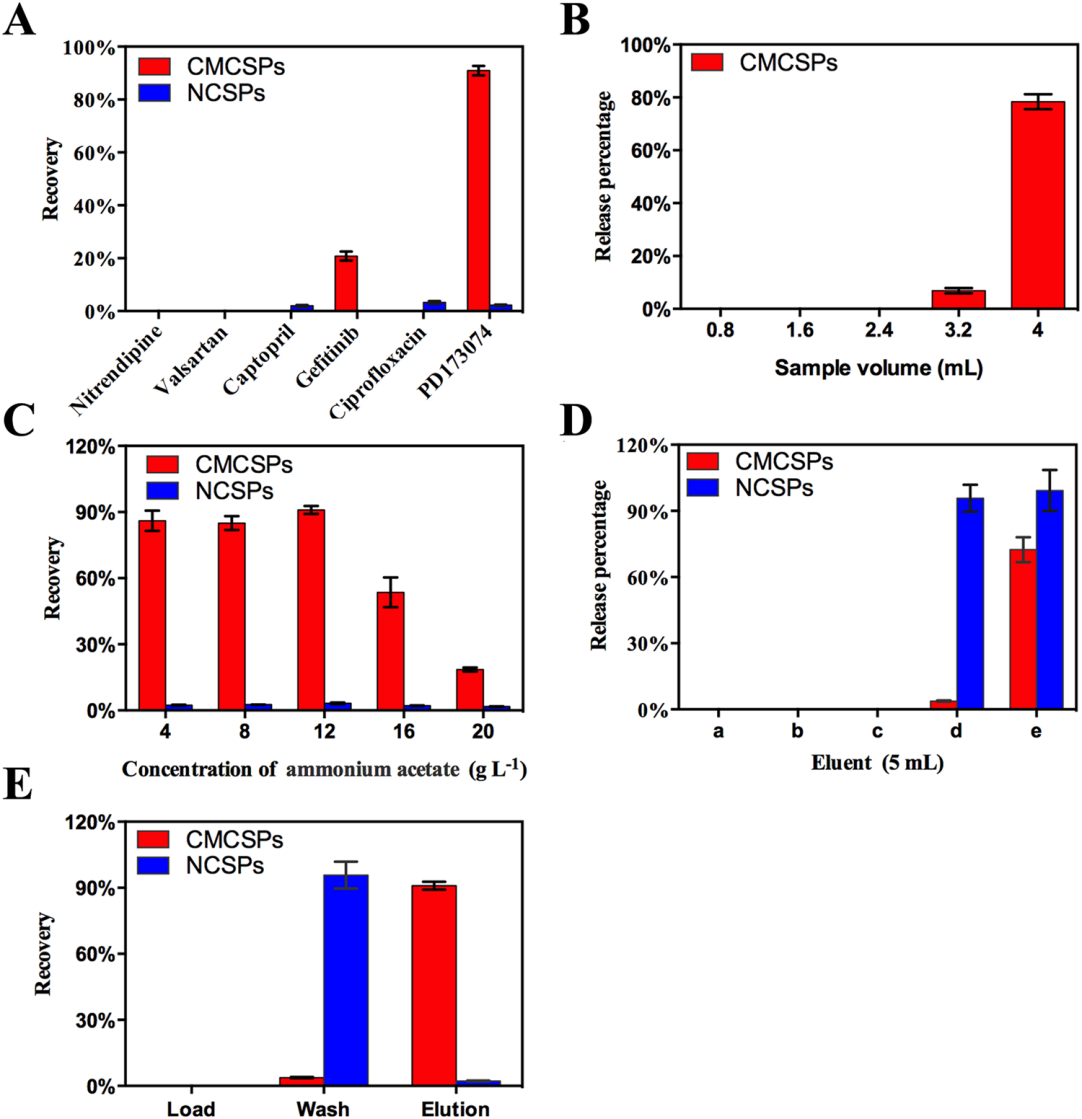
(**A**) Elution recoveries obtained of kinds of medicines acting on different type of receptors; (**B**) Release percentage of PD during the loading step with different sample volume; (**C**) Selection of ionic strength; (**D**) Selection of eluent: water (a), 4 g L^−1^ ammonium acetate buffer solution (b), 12 g L^−1^ ammonium acetate buffer solution (c), isopropyl alcohol-12 g L^−1^ ammonium acetate buffer solution (1:99, v/v) (d) and isopropyl alcohol-12 g L^−1^ ammonium acetate buffer solution (5:95, v/v) (e); (**E**) Recoveries obtained from FGFR4/CMCSPs SPE and NCSPs SPE of PD (5 μg mL^−1^).

The selectivity of FGFR4/CMCSPs is important in the evaluation of SPE sorbents, and was determined using the optimal FGFR4/CMCSPs SPE protocol as follows. The recovery of different drugs which act on different types of receptors are shown in Fig. 4A. The high recovery of PD indicated that it was almost completely adsorbed onto the FGFR4/CMCSPs. The poor recovery of the other drugs on FGFR4/CMCSPs which act on different types of receptors demonstrated that FGFR4/CMCSPs had highly selective binding affinity for compounds acting on FGFR4. In addition, the recovery of all types of drugs on NCSPs SPE was low, which was attributed to poor non-specific adsorption during the washing step. Thus, the FGFR4/CMCSPs showed better selectivity than NCSPs.

### Optimization for FGFR4/CMCSPs in SPE

In order to obtain a more effective FGFR4/CMCSPs SPE protocol, the main extraction parameters were optimized. First, sample volume is an important factor in the loading step. Here, we used isopropyl alcohol-12 g L^−1^ of ammonium acetate buffer solution (1:99, v/v) as the loading solvent, which was used for the binding experiments. PD dissolved in isopropyl alcohol-12 g L^−1^ ammonium acetate buffer solution (1:99, v/v) (5 μg mL^−1^) at different volumes of 0.8, 1.6, 2.4, 3.4 and 4.0 mL was investigated during the loading step to determine the best loading volume. The effluents were collected and analyzed by HPLC. From Fig. 4B, it can be seen that the best loading volume was 2.4 mL, which guaranteed complete adsorption of the sample.

Ionic strength was a vital parameter during the FGFR4/CMCSPs SPE procedure as osmotic pressure caused by saline solution influenced bioactivity of FGFR4 receptors. The effect of ionic strength was characterized in ammonium acetate buffer solution at the concentration range of 4–20 g L^−1^. Figure 4C shows that when the concentration of ammonium acetate was 12 g L^−1^, the recovery of FGFR4/CMCSPs was maximum. These results revealed that 12 g L^−1^ ammonium acetate may be able to provide the cell membrane with a biochemical isotonic condition which could simulate the natural environment of receptors *in vivo*. Thus, 12 g L^−1^ ammonium acetate was used in the experiments.

The washing step aimed to decrease non-specific interactions between the analyte and the materials. In order to achieve a fast wash, a little isopropyl alcohol was used as an organic modifier to reduce non-specific interactions^41, 42^. To better characterize the effect of washing and eluting, the specific parameter *α* was calculated for different solvents. The *α* value was determined as the ratio between the release percentage (%) of NCSPs and FGFR4/CMCSPs columns, which represented the recognition ability of the specific interaction. Theoretically, when *α* was close to 1, the eluent had poor specific recognition ability. In contrast, when *α* was greater than 1, the eluent could eluted impure substances but had little influence on the specific interaction. Figure 4D shows that 5.0 mL isopropyl alcohol-12 g L^−1^ ammonium acetate buffer solution (1:99, v/v) met the standards of the washing solvent whose *α* value was 23.93, which contributed to the suppression of non-specific interactions without disrupting the selective interactions between the active compounds and the cell membrane receptors.

The final elution step was performed to completely remove the captured compounds from FGFR4/CMCSPs, which was the key process in the SPE procedure. In order to achieve good elution efficiency, five eluents including water, 4 g L^−1^ ammonium acetate buffer solution, 12 g L^−1^ ammonium acetate buffer solution, isopropyl alcohol-12 g L^−1^ ammonium acetate buffer solution (1:99, v/v) and isopropyl alcohol-12 g L^−1^ ammonium acetate buffer solution (5:95, v/v) were investigated. Figure 4D demonstrates that water, 4 g L^−1^ ammonium acetate buffer solution and 12 g L^−1^ ammonium acetate buffer solution did not remove the positive drug at all but isopropyl alcohol-12 g L^−1^ ammonium acetate buffer solution (5:95, v/v) had the highest removal efficiency. The adoption of a little organic solvent made it easier to remove the lipophilic components bonded to receptors which could not be eluted by polar solvent^42^. Thus, isopropyl alcohol-12 g L^−1^ ammonium acetate buffer solution (5:95, v/v) was used as the eluent and resulted in satisfactory separation.

### Evaluation of FGFR4/CMCSPs SPE Capacity

In order to evaluate the capacity of the FGFR4/CMCSPs columns, 2.4 mL PD prepared in isopropyl alcohol-12 g L^−1^ ammonium acetate buffer solution (1:99, v/v) was loaded on the FGFR4/CMCSPs and NCSPs cartridges under the above optimized conditions, respectively. From Fig. 4E, it can be seen that the recovery of FGFR4/CMCSPs SPE was 90.93%, which was much higher than the recovery of NCSPs SPE which was 2.27%. Thus, we can conclude that the washing step was suitable for reducing non-specific interactions but retained the active components bonded to the receptor, and guaranteed high binding capacity. In addition, isopropyl alcohol-12 g L^−1^ ammonium acetate buffer solution (5:95, v/v) showed good elution ability.

### Method Validation

The parameters of this method were investigated under the above optimum conditions. The regression coefficients (*r*) obtained and the concentration linear range are shown in Table 3. It was found that the calibration satisfied linearity with a regression coefficient of 0.9999.

**Table 3:** Calibration Graph Parameters, Limits of Detection (LOD), Recoveries (R) and Reproducibility (RSD, *n* = 3) were Calculated from Extractions with Three FGFR4/CMCSPs Columns from Different Batches.

	Calibration graph parameters		LOD (μg mL^−1^)	Recoveries and reproducibility					
	Concentration range (g L^−1^)	Regression coefficient (*r*)		R^a^ (%)	RSD^a^	R^b^ (%)	RSD^b^	Rc (%)	RSD^c^
PD	0.0005–1	0.9999	0.5	77.8	12.8	89.9	7.6	91.0	9.8

^a^Spiked at 0.005 mg mL^−1^.

^b^Spiked at 0.1 mg mL^−1^.

^c^Spiked at 0.5 mg mL^−1^.

The standard addition method was used to evaluate the recovery and reproducibility of the FGFR4/CMCSPs SPE procedures with analyses performed in triplicate. As shown in Table 3, the recovery of spiked PD for extracts from ASG ranged from 77.8% to 91.0%. The corresponding relative standard deviation (RSD) values ranged from 7.6% to 12.8%, which demonstrated that the proposed method had acceptable repeatability.

In addition, the reusability of the absorbents was an important parameter in evaluating application of the proposed method. Here, we used the recovery of PD to investigate reusability of the FGFR4/CMCSPs SPE. The results indicated that the FGFR4/CMCSPs SPE with HPLC method had good repeatability and could be used at least three times without an obvious reduction (RSD < 8.0%). The reproducibility of the different FGFR4/CMCSPs SPE columns was tested using PD standard solutions. The results demonstrated that the RSD of recovery was 15.6% when using different columns (*n* = 3). The precision between different columns met the test requirements.

### Application of FGFR4/CMCSPs in TCM Samples

**Figure 5:**
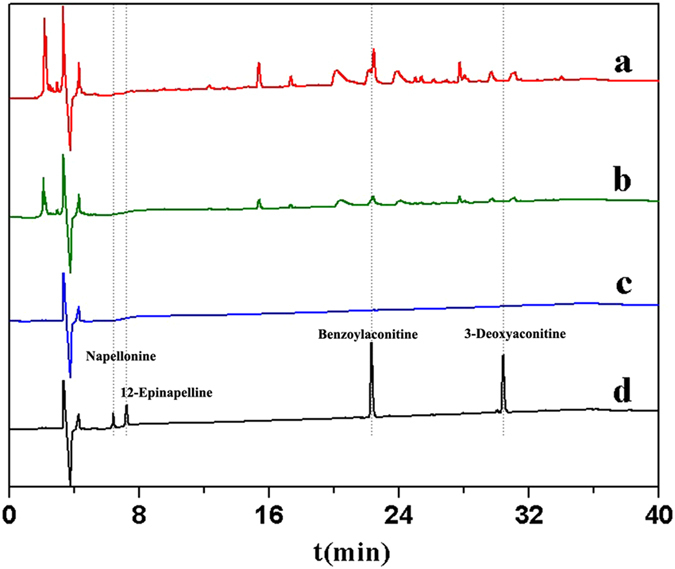
Chromatograms of the extracts from ASG using FGFR4/CMCSPs SPE-HPLC/MS. Initial solutions before FGFR4/CMCSPs SPE (**a**), solution after loading (**b**), solution after washing (**c**) and solution after eluting (**d**).

The proposed method aimed to provide a simple, fast, selective and enriching technique for sample pretreatment, which can be used for screening active compounds from TCM. Figure 5 shows the results of ASG extracts using FGFR4/CMCSPs SPE. Figure 5a is the chromatogram of the components extracted from ASG. Most compounds were not absorbed but passed through the column during the loading step (Fig. 5b). As shown in Fig. 5c, a few constituents were washed out by isopropyl alcohol-12 g L^−1^ ammonium acetate buffer solution (1:99, v/v). Figure 5d is the chromatogram of the bioactive compounds eluted from cell membrane receptors under the experimental conditions and shows that four peaks were observed in these complicated components. Some peaks with high response values in Fig. 5a were not detected in Fig. 5d. Notably, some low-content compounds appeared in the bioactive compounds screened. From these findings, the use of FGFR4/CMCSPs for screening and enriching compounds was based on the specific functional adsorption, not on the concentration of components in TCM.

**Figure 6:**
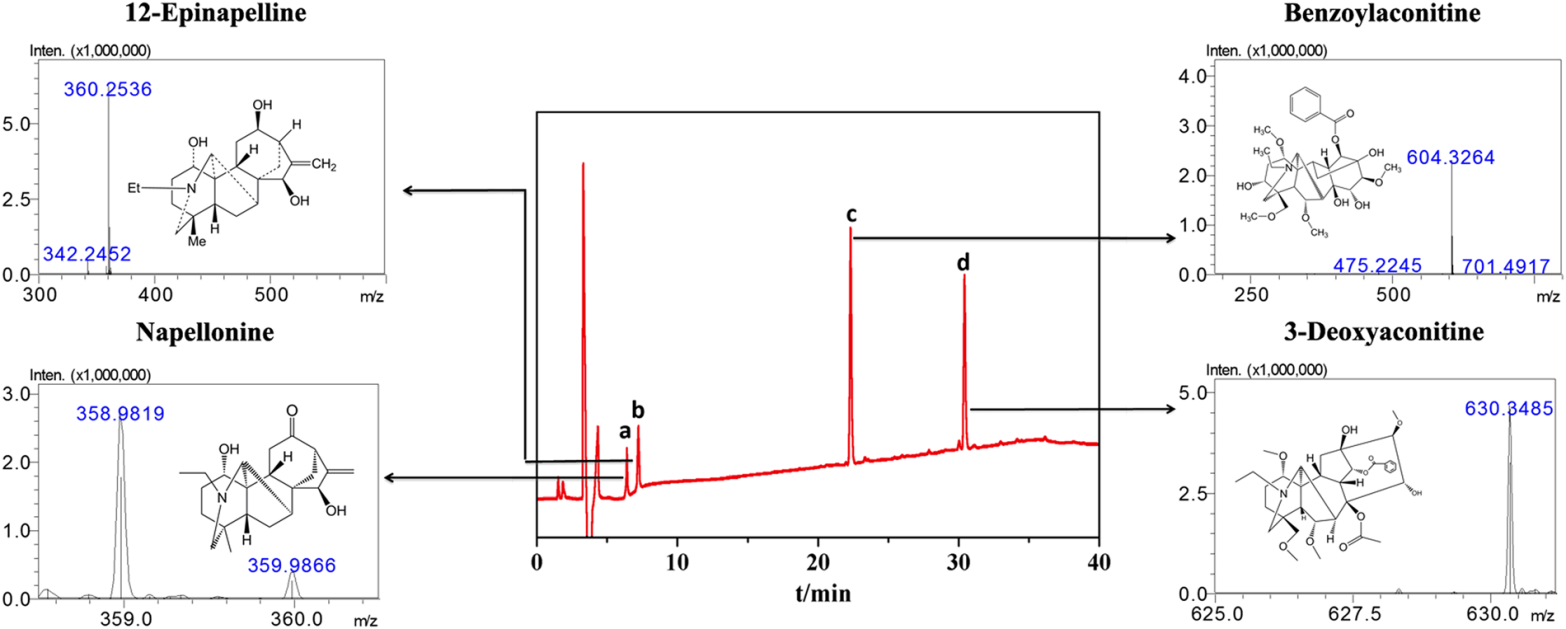
HPLC/MS chromatograms of eluent fraction and main four retention peaks (**a**, **b**, **c** and **d**) were identified as napellonine, 12-epinapelline, benzoylaconitine and 3-deoxyaconitine, respectively.

The retained four peaks were further analyzed by mass spectrometry. Figure 6 shows that the molecular ion peak a of *m*/*z* 358.9819 ([M+H]^+^) was observed and was preliminarily concluded to be napellonine. In order to confirm the structure of the proposed substance, we analyzed the chromatogram and mass spectrum of standard napellonine, and the results were consistent with the above mentioned peak. The analyses of peak b–d are shown in Fig. 6. In conclusion, we identified the other three retention components as 12-epinapelline, benzoylaconitine and 3-deoxyaconitine, respectively. These results demonstrated that the FGFR4/CMCSPs SPE method had the ability to build a screening and enriching active components model from complicated samples.

### Inhibitory Effects of the Screened Alkaloid

**Figure 7:**
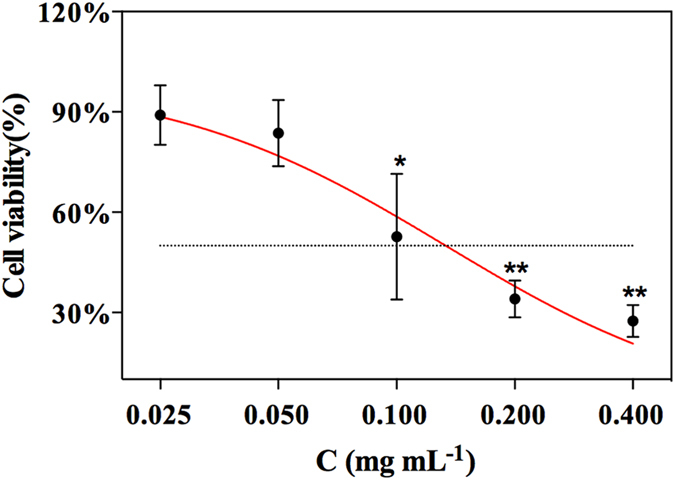
The MTT assay for cell variability on FGFR4 cell. Each value represented the mean ± SD, *n* = 3. Differences between groups were assessed by one-way ANOVA, *p < 0.05, **p < 0.01 vs. control group.

To identify the biological activities of the four screened alkaloid, they were further investigated using the 3-(4,5-dimethylthiazol-2-yl)-2,5-diphenyl-tetrazolium bromide-formazan (MTT) test. Napellonine inhibited cell growth in a concentration-dependent manner (Fig. 7), but the other alkaloid did not inhibit cell growth and may not be FGFR4 antagonists even though they interacted with FGFR4 receptors. The half maximal inhibitory concentration (IC_50_) of napellonine was approximately 0.133 mg mL^−1^ suggesting that napellonine still requires structural modification to achieve a satisfactory clinical effect as a leading compound compared to the IC_50_ value of PD which was 0.0713 mg mL^−1^. However, the results showed that with the addition of the cell proliferation assay FGFR4/CMCSPs SPE is a powerful method to fast screen and enrich bioactive components from complex samples based on the recognition of FGFR4 receptors on the cell membrane.

### Interaction Simulations of the Screened Alkaloid with FGFR4

**Figure 8:**
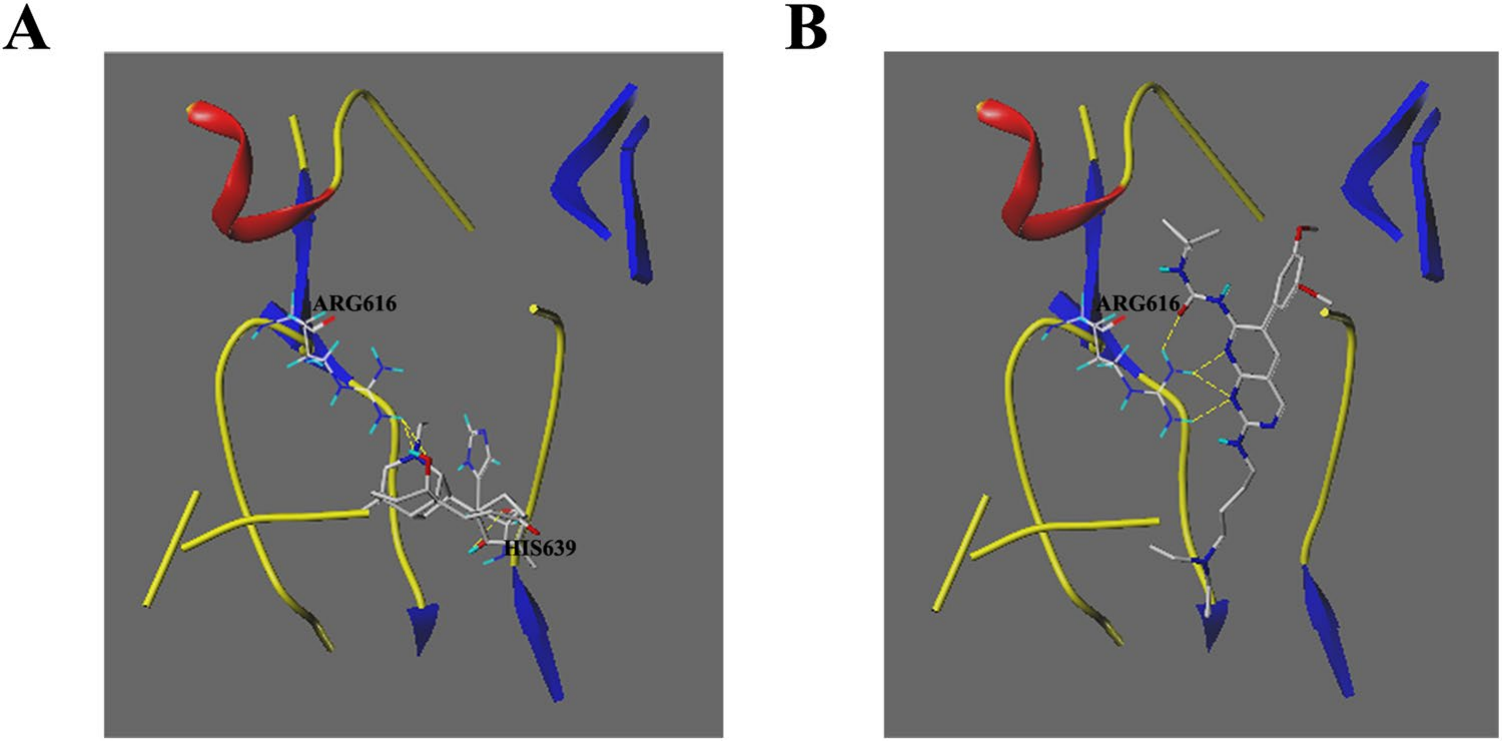
Binding models of (**A**) napellonine and (**B**) PD with FGFR4 (PDB ID: 4TYE). Napellonine and PD were colored based on atom types (carbon: gray; oxygen: red; nitrogen: blue). Yellow dotted lines stood for hydrogen bonds, The key residues were marked in blue.

To determine the possible binding models, docking studies of napellonine, 12-epinapelline, 3-deoxyaconitine and benzoylaconitine with FGFR4 were conducted using Sybyl-X 2.0. The possible binding model of napellonine with FGFR4 is shown in Fig. 8 and the binding models of the other three compounds are displayed in Figure S1, Supporting Information. It showed that napellonine (Fig. 8A) was bound to FGFR4 receptors and displayed a similar binding conformation to that of PD (Fig. 8B). The other three compounds showed different binding models to that of PD (Figure S1), which explained their failure to inhibit cell growth. Napellonine bound to the target protein by three hydrogen bond interactions involving the backbone NH of Arg-616, the side chain OH of Arg-616 and the side chain OH of His-639. It can be concluded that the docking results demonstrated the interaction and geometric fit of the binding model which may provide useful information for napellonine derivatives, based on structural design, as effective inhibitors of FGFR4.

## Conclusions

FGFR4/CMCSPs were successfully prepared using silica particles coated with FGFR4 cell membrane as selective affinity sorbents for screening and preconcentrating bioactive compounds from complex samples. Characterization of the obtained materials by SEM, EDS, TGA and DSC exhibited desired morphological and spectroscopic features which proved that FGFR4/CMCSPs were successfully prepared. FGFR4/CMCSPs SPE showed high adsorption capacity and selectivity to PD, which was adopted as a positive control acting on FGFR4. It was demonstrated that FGFR4/CMCSPs had satisfactory recovery of the positive drug (90.93%), which showed its suitability for screening and enriching active components from ASG. Four compounds were screened and identified, and one of these, napellonine, was shown to have anti-tumor activity by MTT with an IC_50_ of 0.133 mg mL^−1^. In addition, a molecular docking assay showed the possible binding model of napellonine, whose binding conformation to the target protein was similar to PD compared to the other three alkaloid. In summary, the prepared FGFR4/CMCSPs and the SPE procedure showed good application in fast screening and pretreatment bioactive components from TCM.

In the discovery of sorbents for SPE, hundreds of novel sorbents have been prepared and applied for different uses. The proposed CMCSPs can be used as a preliminary screening tool for discovering active leading compounds from TCM or other complex medicine systems. In addition, they can be applied for the preconcentration of micro constituents which cannot be directly analyzed by HPLC. This will be a useful sample pretreatment technique with the dual-function of capture and enrichment of active compounds in natural medicinal herbs.

## Methods

### Materials and Regents

12-Epinapelline, 3-deoxyaconitine, benzoylaconitine and napellonine were from Nanjing SenBeiJia Biological Technology (Jiangsu, China). Nitrendipine, valsartan, captopril, gefitinib and ciprofloxacin were purchased from Nanjing Ange Pharmaceutical Co., Ltd. (Jiangsu, China). PD173074 was from Lanmu Chemical Technology (Shanghai, China). Ammonium acetate and acetic acid were obtained from Fuchen Chemical Agent Co., Inc. (Tianjin, China). Acetonitrile and methanol (HPLC-grade) were purchased from Fisher Scientific (Pittsburgh, USA). All aqueous solutions were prepared using ultrapure water which is produced by MK-459 Millipore Milli-Q Plus ultra-pure water system (Shandong, China). MTT, dulbecco’s modified eagle medium (DMEM) and trypsin were obtained from Sigma (Saint Louis, USA).

### Instrumentation

The obtained FGFR4/CMCSPs and NCSPs particles were fully characterized by SEM, EDS, TGA and DSC. SEM image was obtained via Hitachi S-4800 field-emission SEM equipped with an EDS detector operating at 15.0 kV (Tokyo, Japan). TGA and DSC results were obtained by STA 449 F3 Jupiter (Selb, Germany). The HPLC/MS system (Shimadzu Corporation, Kyoto, Japan) included a SPD-M20A diode array detector (DAD) and a TOFMS system. Sample volume of 10 μL was analyzed at a flow rate of 1.0 mL min^−1^ monitoring at 236 nm for PD. The analytical column was a 150 mm × 4.6 mm, 5 mm C_18_ column (Shimadzu Corporation, Kyoto, Japan). The according mobile phase is consisted of acetonitrile-water-ethanoic acid (70:30:0.5, v/v/v). ASG and its alkaloids were analyzed by 250 mm × 4.6 mm, 5 mm C_18_ column (Acchrom Corporation, Liaoning, China) monitoring at 220 nm. Mobile phase A: acetonitrile, Phase B: 0.1% aqueous formic acid; gradient: 0–30 min, 5% A–30% A; 30–40 min, 30% A–30%A.

### Cell Culture and Preparation of the Standard Solutions

FGFR4 cells were constructed previously in our laboratory^43^. The cell culture medium was made up of DMEM supplemented with 10% fetal bovine serum and 100 μg mL^−1^ of streptomycin. The cells were cultured at 37 °C, 5% CO_2_.

Standard solutions of nitrendipine, valsartan, captopril, gefitinib, ciprofloxacin, PD, 12-epinapelline, 3-deoxyaconitine, benzoylaconitine and napellonine (50.0 mg mL^−1^) were separately prepared in methanol.

### Preparation of FGFR4/CMCSPs

Cells were harvested and washed using physiological saline (pH = 7.4) by centrifugation at 1000 × g and 4 °C for 10 min. The washing procedure was repeated three times to remove residual medium. Following resuspension in 50 mmol L^−1^ Tris–HCl (pH = 7.4), the cells were ruptured by ultrasonication for 30 min, and centrifuged at 2500 × g and 4 °C for 10 min. The supernatant was collected and centrifuged at 12,000 × g and 4 °C for 20 min. The crude membrane precipitate was resuspended in 10 mL physiological saline and centrifuged again at 12,000 × g and 4 °C for 20 min. The precipitate was suspended in 1.0 mL physiological saline. Silica was activated at 120 °C for 30 min, and then added to the cell membrane suspension slowly under vacuum at 4 °C with agitation. The FGFR4/CMCSPs were then obtained. The NCSPs were prepared in the same way as the FGFR4/CMCSPs but without addition of the cell membrane suspension. The concentration of cell membrane proteins in the suspension before and after binding were determined by the bicinchoninic acid protein assay reagent. The amount of adsorbed cell membrane protein immobilized on the silica surface was determined by subtracting the amount of free cell membrane protein in the suspension from the amount of original cell membrane protein. The adsorption efficiency was determined by the ratio of adsorbed to original cell membrane protein. The assay was performed in triplicate.

### Binding Experiments

Following the adsorption experiments were performed in order to evaluate the recognition properties of CMCSPs relative to NCSPs. All assays were conducted in triplicate.

Static adsorption experiments were carried out as follows: a range of different concentrations of 1.0 mL PD (20.0, 50.0, 80.0, 100.0, 500.0, 1000.0, 1500.0, 2000.0, 4000.0, 5000.0, 8000.0 mg L^−1^) were diluted in a mixture of isopropyl alcohol-12 g L^−1^ ammonium acetate buffer solution (1:99, v/v), and then 5.0 mg FGFR4/CMCSPs or NCSPs was added to each sample. The sample tubes were sealed and shaken for 2 h at 37 °C. The mixtures were then centrifuged at 1000 × g and 4 °C for 5 min. The obtained supernatant was evaporated to dryness and redissolved in 0.5 mL methanol. The final concentration of PD was determined by HPLC. The adsorption amounts of the sorbents were calculated by subtracting the residual amounts of PD from the original amounts.

In the adsorption kinetics experiments, FGFR4/CMCSPs or NCSPs (5.0 mg) were added to PD solution (1.0 mL, 150.0 mg L^−1^) and incubated at 37 °C for the following time intervals: 0, 1, 4, 8, 12, 16 and 20 min, respectively. After incubation, the same procedures were performed according to the static adsorption experiments.

In the selectivity experiments, a series of standard solutions (2.4 mL, 5 μg mL^−1^) containing nitrendipine, valsartan, captopril, gefitinib and ciprofloxacin were prepared and loaded onto the FGFR4/CMCSPs or NCSPs cartridge, respectively. The cartridge was then washed with a mixture of isopropyl alcohol-12 g L^−1^ of ammonium acetate buffer solution (1:99, v/v) to eliminate molecules retained by non-specific interactions with the materials, and elution of the analytes was then performed using 5.0 mL isopropyl alcohol-12 g L^−1^ of ammonium acetate buffer solution (5:95, v/v). The collected loading solution, washing solution and elution solution were evaporated and redissolved in 0.5 mL methanol before HPLC analysis.

### SPE Procedures

An empty SPE cartridge (1 mL total volume, 6 mm diameter) was used as the FGFR4/CMCSPs or NCSPs column and was packed with wet filling between two pieces of cotton. As schematically depicted in Fig. 1, 100 mg FGFR4/CMCSPs or NCSPs were added to the SPE column, after being activated by 5.0 mL ultra-pure water, the 2.4 mL PD (5 μg mL^−1^) solution was loaded. The cartridge was then washed with a mixture of 5 mL isopropyl alcohol-12 g L^−1^ of ammonium acetate buffer solution (1:99, v/v). Next, each cartridge was eluted with 5 mL isopropyl alcohol-12 g L^−1^ of ammonium acetate buffer solution (5:95, v/v). The eluent was then evaporated and the residues were redissolved in 0.5 mL methanol for HPLC/MS analysis.

### Extraction of Bioactive Compounds from ASG

ASG was obtained from the Xi’an medicine market (Xi’an, China). The roots of ASG were ground into a powder, 20.0 g of which was then refluxed in 120.0 mL ethanol (60%, v/v) for 2 h to obtain more constituents. The extraction procedure was repeated three times to obtain the optimized extract and the total mixed extract was filtered using a Buchner funnel. The filtrate was then concentrated to a dark brown viscous mass and the extract was then dissolved with 10 mL methanol as the sample.

A 5 mL sample was evaporated to dryness and redissolved in 2.4 mL isopropyl alcohol-12 g L^−1^ of ammonium acetate buffer solution (1:99, v/v). The obtained solution was loaded on the conditioned FGFR4/CMCSPs or NCSPs cartridge. Similarly, the collected loading solution, washing solution and elution solution were evaporated and redissolved in 0.5 mL methanol before HPLC/MS analysis.

### Cell Growth Assay

The effects of 12-epinapelline, 3-deoxyaconitine, benzoylaconitine and napellonine on FGFR4 cell viability were evaluated using the MTT assay. Briefly, FGFR4 cells in the exponential growth phase were harvested, plated in a 96-well plate at a concentration of 5000 cells well^−1^ and incubated for 24 h at 37 °C. The FGFR4 cells were incubated with various concentrations of 12-epinapelline, 3-deoxyaconitine, benzoylaconitine and napellonine for 24 h, respectively. Then, 20 μL MTT (5.0 mg mL^−1^) was added to each well and incubated for 4 h at 37 °C. After discarding the supernatant, 150 μL dimethyl sulfoxide (DMSO) was added to each well to determine absorbance values at 490 nm using a microplate reader (Bio-Rad Instruments, CA, USA).

### Molecular Docking Study

Molecular docking assays were carried out to investigate the possible mechanisms of the anti-tumor effect of napellonine and other compounds. Therefore, protein-ligand docking calculations at the molecular level were performed using Surflex-Dock Module of Sybyl-X 2.0 to identify the underlying binding modes. The X-ray crystal structure of the kinase domain of FGFR4 (PDB ID: 4TYE) was retrieved from the Protein Data Bank. The water molecules were removed. All hydrogen atoms were added. The compounds (napellonine, 12-epinapelline, 3-deoxyaconitine and benzoylaconitine) were constructed and optimized using Powell’s method. Energy minimization was carried out using the Tripos force field with the 0.05 kcal (Åmol)^−1^ convergence criterion and Gasteiger–Hückel charges. The optimized conformations were then docked into the rigid receptor protein using default docking parameters.

## Electronic supplementary material

Supplementary Figure
