# Supplementary material for: A novel cell membrane affinity sample pretreatment technique for recognition and preconcentration of active components from traditional Chinese medicine
Source: Sci Rep. 2017 Jun 15;7:3569. doi: 10.1038/s41598-017-03709-6 (PMC5472601; doi:10.1038/s41598-017-03709-6)
Supplement: Supplementary file 1 — Supplementary Figure [file 41598_2017_3709_MOESM1_ESM.pdf]

Supplementary Figure

A novel cell membrane affinity sample pretreatment technique for recognition and preconcentration of active components from traditional Chinese medicine

Yusi Bu[1,2], Xiaoshuang He[1,2], Qi Hu[1,2], Cheng Wang[1], Xiaoyu Xie[1,2,*], and Sicen Wang[1,2,*]

[1]*School of Pharmacy, Health Science Center, Xi'an Jiaotong University, Xi'an 710061, China*

[2]*Shaanxi Engineering Research Center of Cardiovascular Drugs Screening & Analysis, Xi'an, 710061, China*

*Corresponding author. Tel.: +86 29 82656788; fax: +86 29 82655451

E-mail address: xiexiaoyu@xjtu.edu.cn (X. Xie), wangsc@mail.xjtu.edu.cn (S. Wang).

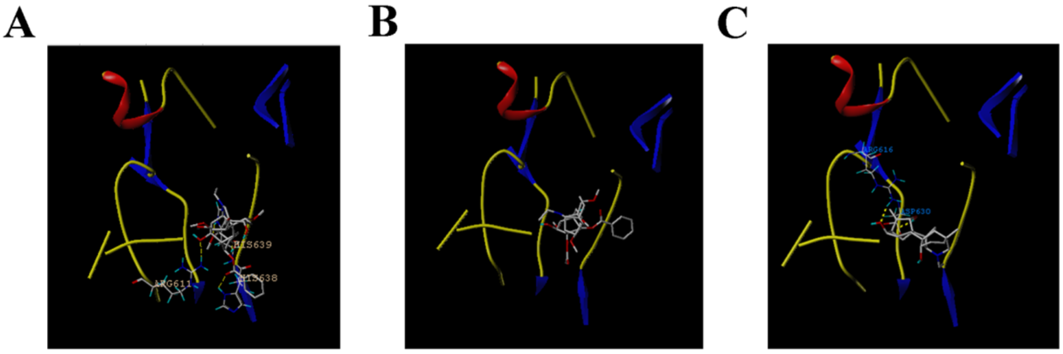

Fig. S1. Binding models of (A) benzoylaconitine; (B) 3-deoxyaconitine and (C) 12-epinapelline with FGFR4 (PDB ID: 4TYE). All alkaloid were colored based on atom types (carbon: gray; oxygen: red; nitrogen: blue). Yellow dotted lines stood for hydrogen bonds, The key residues were marked in blue.
